# *Ginkgo biloba* L. leaf extract (EGb 761) alleviates reserpine-induced depression-like symptoms in aged rats by enhancing serotonin/norepinephrine levels and reducing oxidative/nitrosative stress

**DOI:** 10.1007/s00210-025-03972-9

**Published:** 2025-03-18

**Authors:** Dina H. Ali, Hoda G. Hegazy, Elham H. A. Ali, Hala El-Tantawi

**Affiliations:** 1 Zoology Department, Faculty of Science, Ain Shams University, El-Khalifa El-Mamoun St., Abbassia, Cairo, 11566 Egypt; 2 Zoology Department, Faculty of Women for Arts, Science and Education, Ain Shams University, Cairo, 11757 Egypt

**Keywords:** *Ginkgo biloba* L. leaf extract (EGb 761), Aging, Depression, Reserpine, Monoamines, Synaptophysin

## Abstract

Along with accelerated aging, the prevalence of late-life depression (LLD) exacerbates. Older people are more vulnerable to the adverse effects of antidepressants than the young. Therefore, creating antidepressants from medicinal herbs that are more effective and safer is inevitable. *Ginkgo biloba* L. leaf extract (EGb761) is broadly applied for treating various neuronal dysfunctions. The present study aimed to evaluate the ameliorative and antidepressant effects of EGb761 against reserpine (RES)-induced depression like symptoms and associated comorbidities in aged female rats. Besides, it compared its efficacy with the antidepressant duloxetine (DULX), offering a more comprehensive understanding of therapeutic potential of EGb 761. Rats grouped into control group, EGb 761-H group, RES group, RES plus either EGb 761-L, EGb 761-H, or DULX groups. The antidepressant effects of EGb 761 were evaluated through a series of behavioral tests, measurement of depression biochemical markers, performing neuronal histopathology and immunohistochemical analyses. EGb 761 significantly attenuated behavioral deficits in the open field test and reduced immobility time in the forced swimming test. Moreover, EGb 761 exerted antidepressant-like actions by ameliorating neurotransmitter imbalances, restoring redox homeostasis in cortical region. Also, EGb 761 increased level of ATP, diminished DNA fragmentation, decreased caspase-3 immunoreactivity and increased immunoreactivity of synaptophysin in the cerebral cortex, besides it enhanced the histological architecture of this region. Overall, EGb 761 has the potential effects to manage LLD focus on the role of both serotonergic and noradrenergic systems in mediating these effects, alongside the impact on oxidative/nitrosative stress.

## Introduction

Recently with the acceleration of aging globally, the number of individuals aged 60 years and older will represent 22% of the worldwide population by 2050 according to the World Health Organization (Hopkins et al. 2023). Thus, the incidence of neuropsychiatric disorders among older adults such as depression is expected to grow sharply (Zenebe et al. 2021). Depression in the elderly population is known as late-life depression (LLD). It is the most common mental disorder among older individuals worldwide which affects nearly 10–15% of community-dwelling aged persons (Newman 2016; van Poelgeest et al. 2021). In older patients, depression is a major cause of functional disability and is associated with cognitive deficits and medical comorbidities besides increased risk of dementia and suicide. Moreover, several risk factors are linked to LLD including somatoform disorders, inadequate emotional support and female sex. Women were more likely to experience depression than men (Thorlund et al. 2015; Cheung and Mui 2023). Due to its high prevalence as well as complicated risk factors, LLD is considered as a critical public health challenge. Consequently, its alleviation and enhancement are considered an important sustainable purpose in the maintenance of long-term healthy aging.

Deficiency in monoamine neurotransmitters including serotonin (5-HT), norepinephrine (NE), and dopamine is regarded as the key pathological mechanism in this disorder (Jesulola et al. 2018). Dysregulation of these monoamines in mood-regulating regions of the brain including the cerebral cortex causes depression (Lee and Han 2019). Furthermore, up-regulated oxidative and nitrosative stress besides mitochondrial dysfunction play crucial roles in the development of depression. Brain autopsies and brain imaging studies of depressed patients illustrated the elevation in lipid peroxidation biomarkers (malondialdehyde and carbonyl) and depletion in antioxidant defense, metabolic activity and mitochondrial ATP production (Rossetti et al. 2020; Casaril et al. 2021). Additionally, it was reported that synaptic and neuronal loss in limbic and cortical regions is implicated in the etiology of depression (Cui et al. 2020). Animal models of depression illustrated a reduction in the expression level of synaptic proteins such as synaptophysin which regulates synapse formation and its expression precisely reflects synaptic density (Luo et al. 2021).

The induction of depression in rodents by using reserpine (RES) is a commonly utilized animal model to evaluate the pathological symptoms of depression (Miguel Telega et al. 2024). RES was used as a primary therapy for hypertension; nevertheless, after prolonged usage, it caused depression as a serious side effect. This adverse effect resulted from its ability to deplete brain monoamines (Kim et al. 2021).

Traditional antidepressants are the primary option for pharmacological treatment for older individuals suffering from depression. However, these medications are modestly effective in LLD therapy with a response rate of 51% (Ishtiak-Ahmed et al. 2023). Moreover, older individuals are at increased risk of antidepressant adverse effects due to polypharmacy, the reduction of physiological reserve, and age-related changes in pharmacokinetics and pharmacodynamics (van Poelgeest et al. 2021). The antidepressant duloxetine (DULX) is one of the first-line drugs recommended for older adults as serotonin and norepinephrine reuptake inhibitor (Kuo et al. 2021). Nevertheless, DULX exhibited oxidative effects due to the bioactivation of its naphthyl and thiophene rings (Álvarez-González et al. 2021). Accordingly, finding new antidepressants with high efficacy and safety for the elderly is so crucial.

Herbal medicine has been considered as an alternative therapeutic approach for aging people, because they showed less adverse effects, present cultural acceptability, as well as having reduced costs. Among the medicinal plants which can enhance age-related issues is *Gingko biloba* L. which belongs to family Ginkgoaceae (Dziwenka and Coppock 2016). Leaf extract of *Gingko biloba* L. is one of the best-selling herbal medicines in the world. EGb761 is a commercially available standardized extract of *Ginkgo biloba* L. leaves that contains 22–27% flavonoids and 5–7% terpene lactones (Zhao et al. 2021). The pharmacological properties of these bioactive components include antitumorigenic (Park et al. 2016), antioxidant (Zhou et al. 2017), neuroprotective (Yu et al. 2021), anti-inflammatory (Zhang et al. 2023), and anti-apoptotic activities (Ovey et al. 2023). Due to the diverse therapeutic properties of EGb 761, it has been commonly used to treat cerebrovascular insufficiency, age-related memory deficits, cognition impairment, and dementia (Brüggemann et al. 2021; Nowak et al. 2021). In addition, EGb 761 exhibited antidepressant effects against anhedonia depressive behavior in male rats induced by lipopolysaccharide (Yeh et al. 2015).

While the antidepressant effects of *Gingko biloba* L., particularly EGb 761, have been widely reported, our study offers new insights by focusing on its effects in an aged rat model of depression, which is underrepresented in the existing literature (Dai et al. 2018). Specifically, our research provides a detailed examination of the biological mechanisms through which EGb 761 exerts antidepressant-like effects, alongside its neuroprotective properties, at two different doses. Moreover, we compare the efficacy of EGb 761 with the antidepressant drug DULX, offering a more comprehensive understanding of its therapeutic potential. This study also aims to assess the potential of EGb 761 as a standalone treatment for LLD, with the goal of reducing the medication burden in elderly patients, who often require multiple medications for various health conditions.

## Materials and methods

### Chemicals and drugs

RES (CAS number 50–55-5) was obtained from Sigma-Aldrich company (Missouri, USA). It was dissolved in 1% (v/v) dimethyl sulfoxide (DMSO) that was also purchased from Sigma-Aldrich company. The standardized leaf extract of *Gingko biloba* L. (EGb 761 capsules; Batch number: 211898A) was obtained from Future Pharmaceuticals industries for EMA Pharm (Cairo, Egypt). The extract was adjusted by the manufacturer to contain 24% flavonol glycosides, 6% terpene lactones, as well as 7% proanthocyanidins and organic acids. The major flavonol constituents of the extract are flavonol-O-glycosides of quercetin, kaempferol, or isorhamnetin linked to D-glucose, L-rhamnose, or glucorhamnose. The terpene lactones mainly include ginkgolides, A, B, C, and J which account for 3.1% and bilobalide which account for 2.9% of the total extract. DULX was purchased from Eli Lilly Pharmaceutical Company (Cairo, Egypt). Both EGb 761 and DULX were dissolved in saline (0.9% NaCl). All other chemicals used were highly analytical grade and utilized without further purification.

### Animals

Thirty female Wistar albino rats (*Rattus norvegicus*), 18 months old, were obtained from the animal house of the Egyptian Company for biological products and Vaccines (VACSERA; Helwan, Egypt). Animals were placed in plastic cages at controlled temperature of 23 ± 2 °C and relative humidity of 45 ± 5% under 12-h/12-h light/dark cycle. Rats acclimatized to the laboratory conditions for 2 weeks before starting the experiment with free access to pellet diet and water. During the acclimatization period, daily vaginal cytology was performed to ensure that all aged female rats included in our study were in the same reproductive phase. The animals were humanely treated in compliance with the National Institutes of Health Guide for the Care and Use of Laboratory Animals. The research protocol was approved by Research Ethics Committee of Faculty of Science, Ain Shams University (Code: ASU-SCI/ZOOL/2024/10/7).

### Experimental design

Thirty aged female rats were randomly assigned to six experimental groups (*n* = 5 per group). Below is a clear breakdown of the experimental design and treatment schedule for each group:

Group 1(Control group): the rats received initial treatment (Day 1–15) as a daily intraperitoneal (i.p.) injection of 1% DMSO (vehicle). Then, they received post-treatment (Day 16–30) as an oral saline (0.9% NaCl) daily, along with i.p. injection of 1% DMSO every other day. A 1-h interval was maintained between oral and i.p. treatments. Group 2 (EGb 761-H group): the rats also received initial treatment (Day 1–15) as a daily i.p. injection of 1% DMSO. After that, they received post-treatment (Day 16–30) as a daily oral administration of high-dose EGb 761 (EGb 761-H; 200 mg/kg/day) (Lee et al. 2002), with i.p. injection of 1% DMSO every other day. A 1-h gap was kept between treatments. Group 3 (RES group; depression induction): the rats received initial treatment (Day 1–15) as a daily i.p. injection of RES (0.2 mg/kg/day) to induce depression (Antkiewicz-Michaluk et al. 2014). After that, they received post-treatment (Day 16–30) as a daily oral administration of saline (0.9% NaCl), along with i.p. injection of RES (0.1 mg/kg) every other day to maintain the depressive state in rats. A 1-h interval was kept between treatments.

**Fig. 1:**
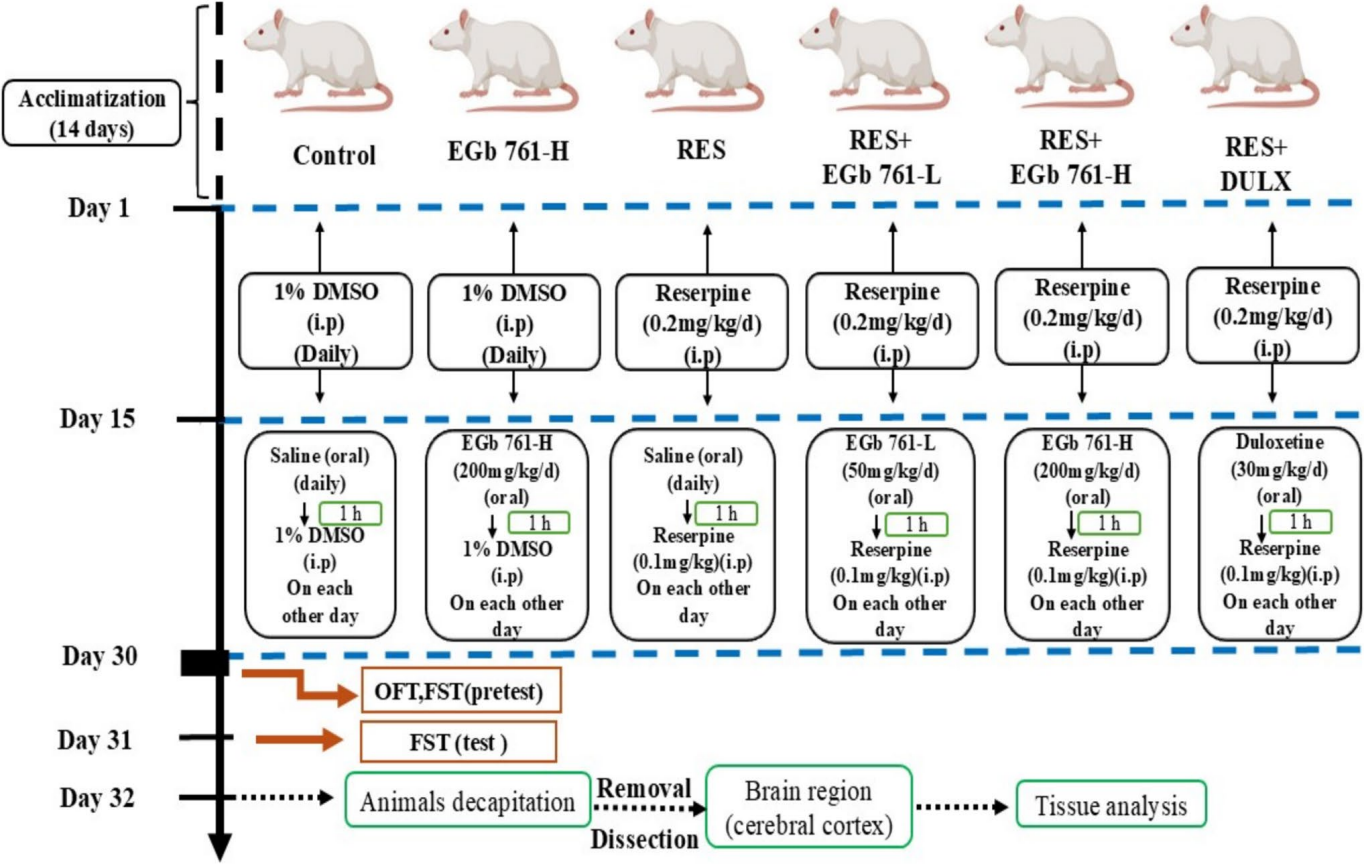
Schematic diagram of the experimental design

Group 4 (RES + EGb 761-L group): the rats received initial treatment (Day 1–15) as a daily i.p. injection of RES (0.2 mg/kg/day). Then, they received post-treatment (Day 16–30) as an oral administration of low-dose EGb 761 (EGb 761-L; 50 mg/kg/day) (Yeh et al. 2015), with i.p. injection of RES (0.1 mg/kg) every other day as a maintenance dose to depressive state in rats. A 1-h interval was kept between treatments. Group 5 (RES + EGb 761-H group): the rats received initial treatment (Day 1–15) as a daily i.p. injection of RES (0.2 mg/kg/day). After that, they received post-treatment (Day 16–30) as an oral administration of high-dose EGb 761 (EGb 761-H; 200 mg/kg/day), along with i.p. injection of RES (0.1 mg/kg) maintenance dose every other day. A 1-h gap between treatments was maintained. Group 6 (RES + DULX group): the rats received initial treatment (Day 1–15) as a daily i.p. injection of RES (0.2 mg/kg/day). Then, they received post-treatment (Day 16–30) as a daily oral administration of DULX (30 mg/kg/day) (Zomkowski et al. 2012), with i.p. injection of RES (0.1 mg/kg) every other day to maintain depression model. A 1-h interval was kept between treatments. After the final injection (Day 30), all groups underwent behavioral testing. Open field test (OFT) and pretest session of forced swimming test (FST) were conducted on day 30. A 2-h interval was kept between 2 behavioral tests. The test session of FST was conducted on day 31. The experimental design diagram is illustrated in Fig. 1.

### Behavioral assessments

#### Open field test (OFT)

OFT was performed to evaluate the locomotor and exploratory behaviors in rats. It was conducted using black wood-made box (100 × 100 × 40 cm) with a floor sectioned into 16 equal sized squares and the middle four squares designated as the central zone. The rats were individually placed at the center of the open-field box and their movements were recorded for 10 min via a camera above the apparatus. The recordings were automatically analyzed using SMART v3.0 video tracking software (Panlab S.L.U, Barcelona, Spain) for two measures including total traveled distance and time spent in the central zone. After each test, the apparatus was wiped with 70% alcohol to avoid confounding odors (Gong et al. 2019).

#### Forced swimming test (FST)

The rats were individually forced to swim in a Plexiglass cylinder (60 cm high, 20 cm diameter) filled with water at 25 ± 2 °C up to 40 cm for 15 min in the pretest session. They were forced again to swim in the cylinder for a 5-min test session 24 h later. The activity of each rat was videotaped, recorded and analyzed off-line with video tracking software; SMART v3.0 (Panlab S.L.U, Barcelona, Spain) to measure the immobility time. Immobility was characterized by the absence of all motion of the rat other than the minimal movements needed to keep its head above the water. After each session, the water in the swim cylinder was entirely changed to bypass any influence on the next rat. The immobility period in FST was recorded to evaluate desperation behavior (Adachi et al. 2022).

### Brain tissue collection and homogenate

Twenty-four hours after FST, rats were sacrificed by rapid decapitation. Brains were quickly removed and immediately put on ice followed by rinsing with ice-cold isotonic saline (0.9% NaCl) to remove the excess blood and the residues. Each brain was sectioned into the right and left halves and then each half was dissected to excise the cerebral cortex. Each the excised cortical tissue of the left halves was fixed in 10% formalin for histopathological and immunohistochemical examination while each one of right halves was weighted and frozen at − 80 °C for further analysis. Parts of frozen cortical samples of each right half were homogenized in 0.05 M cold phosphate buffer saline, pH 7.4 and the other parts were homogenized in lysis buffer to prepare 10% (w/v) homogenates for biochemical and DNA fragmentation assays, respectively.

### Biochemical analysis

#### Quantification of 5-HT, NE and ATP by ELISA

The levels of 5-HT, NE, and ATP in cortical tissue homogenates were detected by using 5-HT and NE ELISA kits (Cat. No: ELK8954 and ELK8317, respectively; ELK. Biotechnology Co. Ltd., Wuhan, China) and ATP ELISA kit (Cat. No: E0920Ra; BT LAB, Shanghai Korain Biotech. Co., Ltd., Zhejiang, China) according to the instructions of manufacturer. The results were shown in micrograms per gram tissue (µg/g tissue).

#### Determination of oxidative and nitrosative stress parameters

The levels of reduced glutathione (GSH) and oxidized glutathione (GSSG) were assessed according to the method described by Van Doorn et al. (1978). This method depends on the reduction of Ellman’s reagent; 5,5′ dithiobis-(2-nitrobenzoic acid) or DTNB by sulfhydryl groups in GSH to form a yellow compound. The absorbance of generated chromogen can be measured spectrophotometrically at 412 nm. According to this concept, GSH content was detected before and after GSSG reduction by using sodium borohydride to determine GSH and total GSH (GSH + GSSG) contents, respectively. Their subtraction from each other gave GSSG content in the samples. GSH and GSSG levels were represented in µmol/g tissue.

The cortical level of malondialdehyde (MDA) was used to estimate the degree of lipid peroxidation according to Esterbauer and Cheeseman (1990) method. Under acidic conditions in water bath at 90 °C for 25 min, one molecule of MDA reacted with two molecules of thiobarbituric acid (TBA) to form thiobarbituric acid reactive substances. The absorbance of the generated pink product was measured using spectrophotometer at 532 nm. MDA levels were expressed in µmol/g tissue.

The nitric oxide (NO) content was measured using Elabscience kit (Cat. No: E-BC-K035-S; Texas, USA) according to the method described by Sun et al. (2003). This method relies on the assessment of endogenous nitrite content as an index of NO production in tissue homogenate. The addition of Griess reagent altered nitrite into a reddish azo compound whose absorbance was measured at 550 nm. NO content, calculated indirectly, was reported as mmol/g tissue.

#### Quantitative assessment of DNA fragmentation

DNA fragmentation in a biological sample homogenized in lysis buffer (5 mM Tris–HCl, 20 mM EDTA, pH 8.0, 0.5% Triton X-100) was assessed according to Gercel-Taylor (2005) method. DNA was completely hydrolyzed in hot acidic conditions into purines and deoxyriboses. The resultant deoxyriboses were oxidized into 5-hydroxy-4-oxopentanals that were dimerized and reacted with diphenylamine specifically to form blue-colored compound whose absorbance can be read at 600 nm. Data was reported as percent fragmented DNA of the total DNA.

### Histopathological examination

The formalin-fixed cortical tissues were routinely processed for embedding in paraplast and sectioned with a microtome at 5 µm thickness. Subsequently, sections were deparaffinized and stained with hematoxylin and eosin (H&E) according to Bancroft and Gamble (2008) methods. Stained slides were examined and photographed using a light microscope (Leica DM750) coupled with Leica ICC50 a high-resolution camera.

### Immunohistochemical detection of caspase-3 and synaptophysin

Immunohistochemical staining was carried out as previously described by Hayat (2002) using Ventana BenchMark XT automated System (Ventana Medical Systems, Tucson, USA). Additional 5-µm-thick paraffin sections were cut, mounted on positive-charged slides, deparaffinized, and rehydrated. Then, they were treated with 3% hydrogen peroxide to block the endogenous peroxidase activity. For epitope retrieval, the slides were rinsed in citrate buffer pH 6 with steam for 1 h followed by incubation with the primary antibodies. Rabbit polyclonal antibodies against caspase-3 (Thermo fisher, USA) and synaptophysin (Abcam, Cambridge, UK) were used at a specified dilution in antibody diluent solution. Bound antibodies were detected utilizing the kit of Ventana UltraView Universal DAB Detection according to manufacturers’ protocols. The sections were counterstained with Mayer’s hematoxylin solution then examined and imaged under × 40 magnification using Leica DM750 light microscope. The percent of immunoreactivity area of caspase-3 and synaptophysin in cortical tissues were analyzed and measured with Fiji-ImageJ Software (v1.51a NIH).

### Statistical analysis

All data were expressed as mean ± standard error of mean (SEM). GraphPad Prism version 8.0.2 for Windows (GraphPad Software Inc; Boston, MA, USA) was utilized for data analysis*.* All experimental values have passed the Shapiro–Wilk normality test. Thus, statistical level of significance analysis was performed using one-way analysis of variance (ANOVA) followed by Tukey’s multiple comparisons test to determine the differences among groups. *p* values of < 0.05, < 0.01, < 0.001, and < 0.0001 were deemed statistically significant, highly significant, very highly significant, and very very highly significant, respectively.

## Results

### EGb 761 ameliorated depression-like behaviors induced by RES in aged rats

**Fig. 2:**
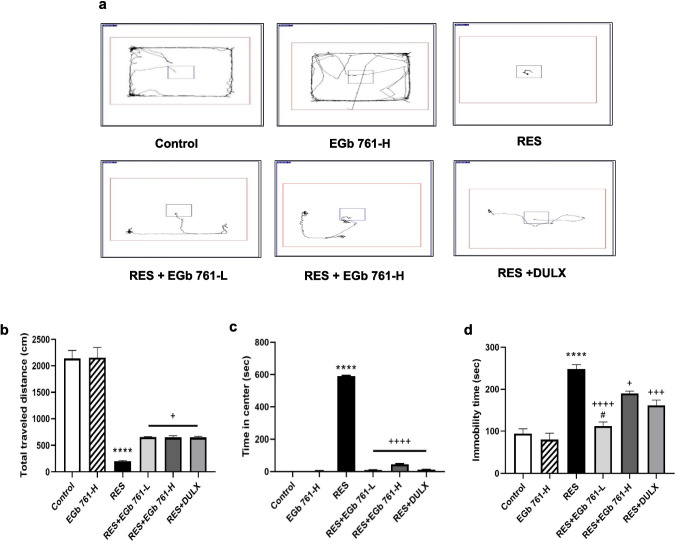
EGb 761 enhanced RES-induced depression-like behaviors in aged rats. **a** Movement trajectory of aged rats in each group, **b** the total traveled distance, and **c** the time in central area in OFT. **d** The immobility time in FST. Values are represented as the mean ± SEM, with *n* = 5. ****p < 0.0001 compared with the control group; ^**+**^*p* < 0.05, ^**+++**^*p* < 0.001, ^**++++**^*p* < 0.0001 compared with the RES group; ^**#**^*p* < 0.05 compared with the DULX group. Statistical analysis was performed by one-way ANOVA followed by Tukey’s multiple comparisons test

The outcomes of OFT analysis in Fig. 2a–c showed that the RES group had a significant decrease in the total traveled distance (− 90.66%), but a marked increase in the central duration (55,626.42%) compared with that in the control group. When compared with the RES group, the treatment of depressed rats with EGb 761-L, EGb 761-H, or DULX could significantly reverse both the reduction in the total traveled distance (226.3%, 224.75%, and 225.4%, respectively) and the increase in the central duration (− 98.31%, − 92.47%, and − 97.98%, respectively). These results indicated that EGb 761 revealed an antidepressant-like effect which is equivalent to that of DULX by ameliorating the loss of spontaneous locomotor behavior and exploratory activity in the aged rats treated with RES.

The outcomes of FST analysis in Fig. 2d illustrated that the immobility duration of RES group was significantly higher (164.97%) than that of the control group. This result revealed that rats injected with RES were more desperate, confirming that RES successfully induced depression-like behavior. Moreover, EGb 761-L, EGb 761-H, or DULX administration to RES-injected animals strikingly shortened the immobility duration (− 54.84%, − 23.33%, and − 34.79%, respectively) in contrast to that in the RES group. Consequently, FST findings indicated that EGb 761-L had the most significant effect to enhance the behavioral despair provoked by RES in aged rats.

### EGb 761 modulated RES-triggered monoamine neurotransmitters imbalance in the cerebral cortex of aged rats

**Fig. 3:**
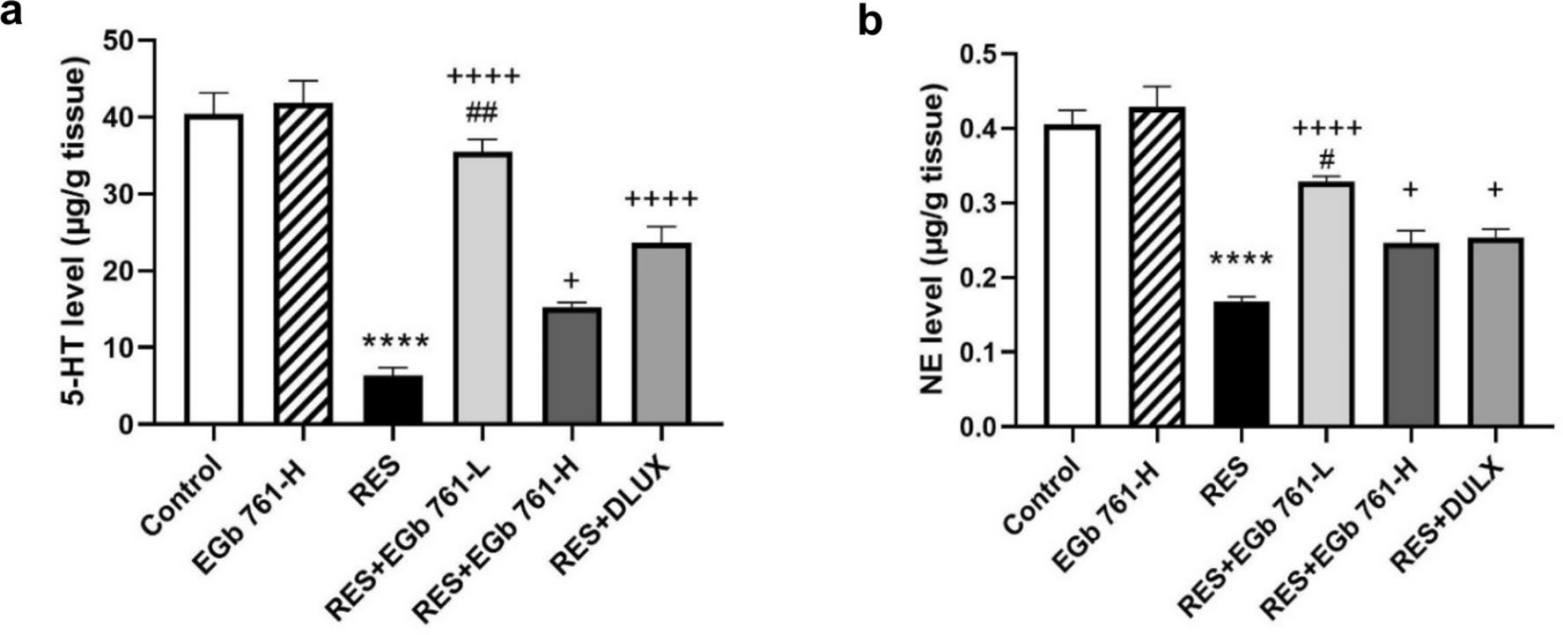
EGb 761 modulated monoamines imbalance induced by RES in cerebral cortex of aged rats. **a** The level of cortical serotonin (5-HT). **b** The level of cortical norepinephrine (NE). Values are represented as the mean ± SEM, with *n* = 5. ****p < 0.0001 compared with the control group; ^**+**^*p* < 0.05, ^**++++**^*p* < 0.0001 compared with the RES group; ^**#**^*p* < 0.05, ^**##**^*p* < 0.01 compared with the DULX group. Statistical analysis was performed by one-way ANOVA followed by Tukey’s multiple comparisons test

The levels of 5-HT and NE were dramatically lower (− 84.21% and − 58.52%, respectively) in rats treated with RES than those in control animals (Fig. 3). In contrast, the depressed rats administered with EGb 761-L, EGb 761-H, or DULX had a remarkable elevation in the levels of 5-HT (456.43%, 138.56% and 270.69%, respectively) relative to those in RES group. In addition, they illustrated a notable rise in NE levels (95.83%,46.43%, and 50.6%, respectively) compared to those in RES group. These results suggested that EGb 761 especially EGb 761-L was more potent (*p* < 0.01) than DULX to modulate both 5-HT and NE concentration in cerebral cortex of RES-injected rats.

### EGb 761 attenuated RES-triggered oxidative and nitrosative stress in aged rats’ cerebral cortex

**Fig. 4:**
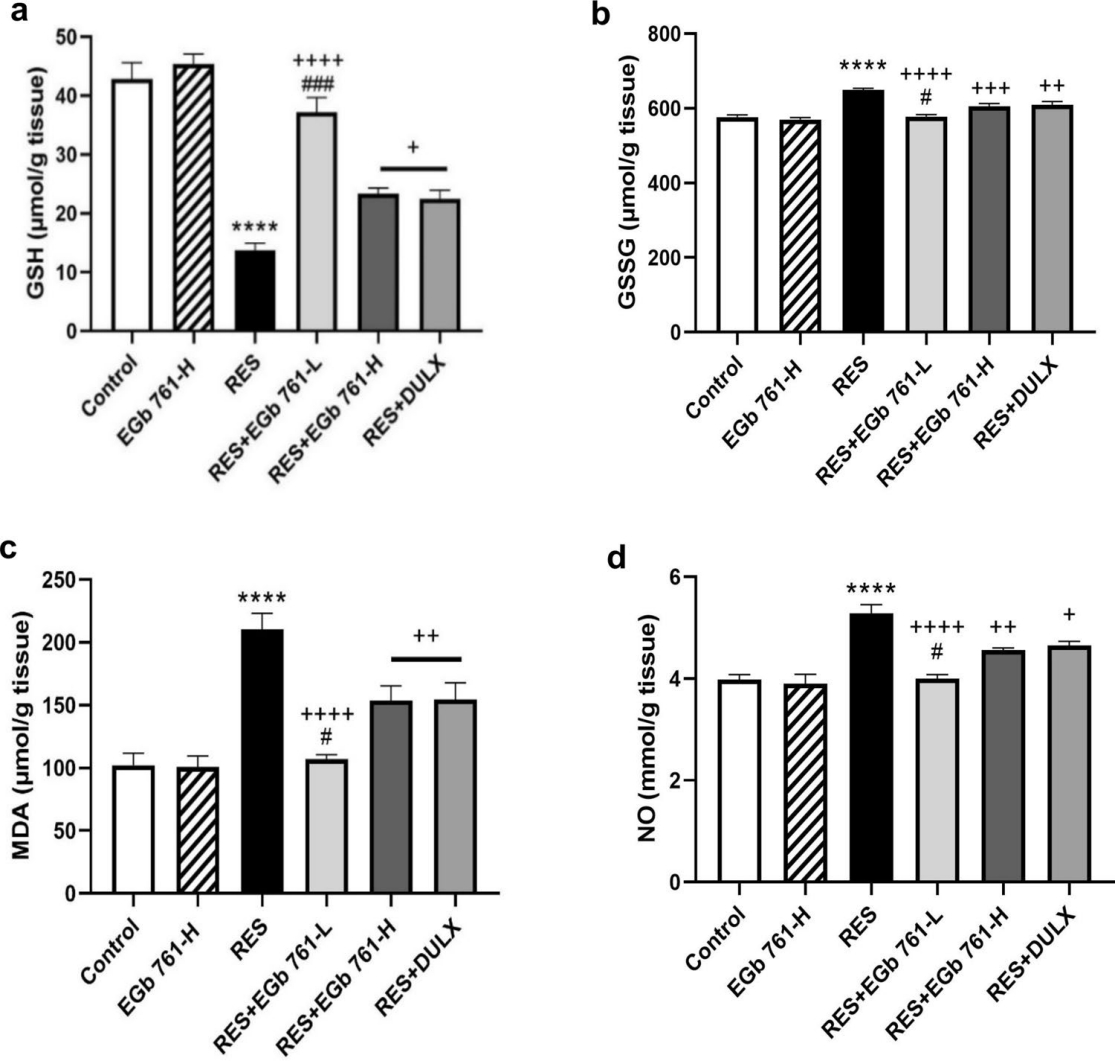
EGb 761 attenuated oxidative and nitrosative stress triggered by RES in cerebral cortex of aged rats. The cortical levels of **a** reduced glutathione (GSH), **b** oxidized glutathione (GSSG), **c** malondialdehyde (MDA), and **d** nitric oxide (NO) were detected. Values are represented as the mean ± SEM, with *n* = 5. ****p < 0.0001 compared with the control group; ^**+**^*p* < 0.05, ^**++**^*p* < 0.01, ^**+++**^*p* < 0.001, ^**++++**^*p* < 0.0001 compared with the RES group; ^**#**^*p* < 0.05, ^**###**^*p* < 0.001 compared with the DULX group. Statistical analysis was performed by one-way ANOVA followed by Tukey’s multiple comparisons test

RES injection caused a robust lowering in cortical level of GSH (− 67.97%) and a significant increment of GSSG, MDA, and NO levels (12.61%, 106.07%, and 32.75%, respectively) compared to the control group (Fig. 4). Contrarily, the treatment of RES- injected animals with EGb 761-L, EGb 761-H, or DULX induced a statistical significant rise in GSH level (170.58%, 69.99%, and 63.8%, respectively) with prominent depletion in the levels of GSSG (− 11.06%, − 6.68%, and − 6.21%, respectively), MDA (− 49.19%, − 27.07%, and − 26.64%, respectively), and NO (− 24.29%, − 13.47%, and − 11.76%, respectively) compared with RES group, suggesting that EGb 761-L was superior in alleviating oxidative and nitrosative stress induced by RES to EGb 761-H and DULX.

### EGb 761 reversed the decline in ATP production in cerebral cortex of depressed aged rats

The content of cortical ATP (the primary energy molecule) was detected by ELISA to investigate EGb 761 effect on cellular energy. As revealed in Table 1, the aged rat model of depression had a smaller content of ATP (− 37.58%) than that of control group. On the other hand, EGb 761-L administration to depressed animals caused a significant elevation in ATP content (45.73%) compared with RES group. However, EGb 761-H and DULX administered to depressed one augmented the content of ATP, but it was statistically insignificant as compared with RES group. The findings illustrated that EGb 761-H and DULX were less effective than EGb 761-L to enhance cellular energy in RES-injected animals.

**Table 1:** EGb 761 reversed the reduction in cortical ATP production in RES-injected rats

	Control	EGb 761-H	RES	RES + EGb 761-L	RES + EGb 761-H	RES + DULX
ATP (µg/g tissue)	27.57 ± 1.68	28.27 ± 1.73	17.21 ± 1.18***	25.08 ± 1.3^**++, #**^	18.2 ± 1.3	18.07 ± 0.94

Values are represented as the mean ± SEM, with *n* = 5. ***p < 0.001 compared with the control group; ^**++**^*p* < 0.01 compared with the RES group; ^**#**^*p* < 0.05 compared with the DULX group. Statistical analysis was performed by one-way ANOVA followed by Tukey’s multiple comparisons test

### EGb 761 declined RES-provoked DNA damage in cerebral cortex of aged rats

**Fig. 5:**
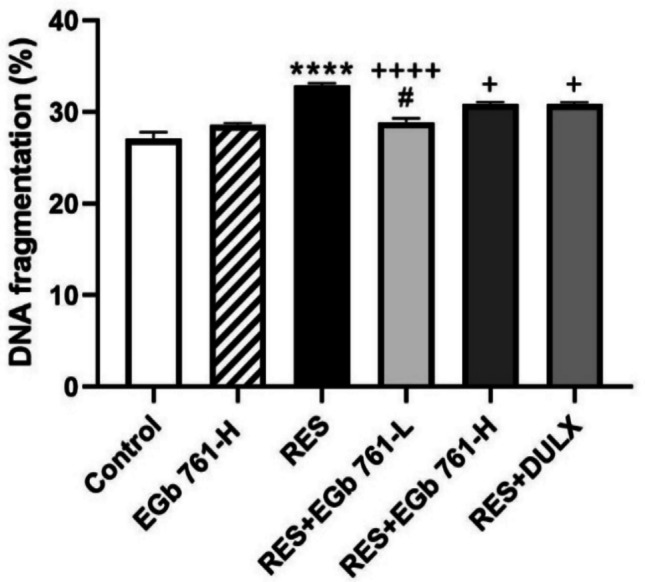
EGb 761 diminished damage of DNA provoked by RES in region of cerebral cortex of aged animals. Values are represented as the mean ± SEM, with *n* = 5. ****p < 0.001 compared with the control group; ^**+**^*p* < 0.05, ^**++++**^*p* < 0.0001 compared with the RES group; ^**#**^*p* < 0.05 compared with the DULX group. Statistical analysis was performed by one-way ANOVA followed by Tukey’s multiple comparisons test

The injection of RES triggered notably exacerbation in DNA fragmentation percentage (21.32%), indicative of an elevation in apoptotic cell death in comparison to control group (Fig. 5). Conversely, EGb 761-L, EGb 761-H, and DULX treatment ameliorated DNA damage in cortical tissue of RES-injected animals by markedly decreasing percentage of DNA fragmentation (− 12.06%, − 6.03%, and − 5.97%, respectively) compared with RES group. Finally, the administration of EGb 761-H to control rats did not exhibited any significant change in DNA fragmentation percentage or any above parameter in relative to control group.

### EGb 761 diminished caspase-3 immunoreactivities in cortical tissue of RES-injected aged rats

**Fig. 6:**
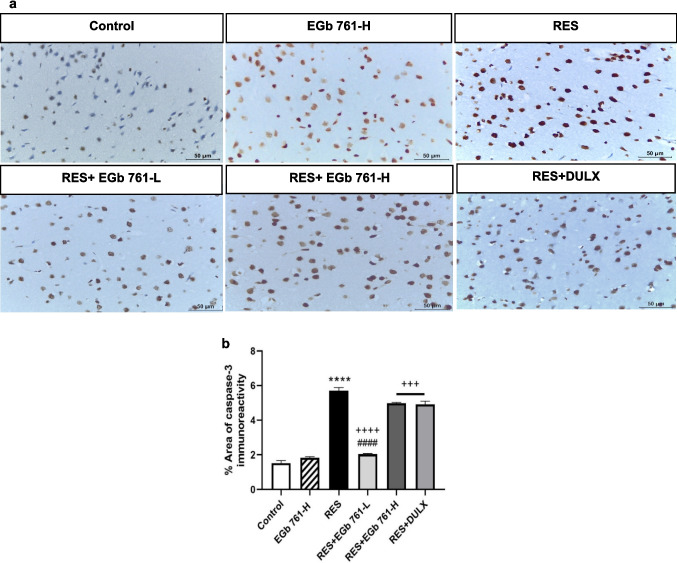
EGb 761 reduced RES-elicited the elevation of caspase-3 immune reaction in cerebral cortex region. **a** Representative immunohistochemistry photomicrographs of caspase-3 expression at scale bar equals 50 µm. **b** Percentage area of caspase-3 immune expression (*n* = 5 per group). Values in bar chart represented as the mean ± SEM. ****p < 0.0001 compared with the control group; ^**+++**^*p* < 0.001, ^**++++**^*p* < 0.0001 compared with the RES group; ^**####**^*p* < 0.0001 compared with the DULX group. Statistical analysis was performed by one-way ANOVA followed by Tukey’s multiple comparisons test

To further explore EGb 761 influence on neuronal apoptosis induced by RES, the immunohistochemical staining was carried out to analyze the expression of caspase-3 as apoptotic marker in cerebral cortex region (Fig. 6). The control group revealed negative caspase-3 immunoreactivity in the cytoplasm of most pyramidal and granular cells, but EGb 761-H group illustrated weak positive immune reaction of caspase-3. Furthermore, the injection of RES induced a significant exacerbation in caspase-3 immunoreactivity (273.67%) compared to the control group. Alternatively, the administration of EGb 761-L, EGb 761-H, or DULX to depressed rats resulted in a remarkable reduction in caspase-3 immune expression (− 64.4%, − 12.82%, and − 14.02%, respectively) in comparison to RES group.

### EGb 761 improved RES-induced histopathological aberrations in cerebral cortex tissue of aged rats

**Fig. 7:**
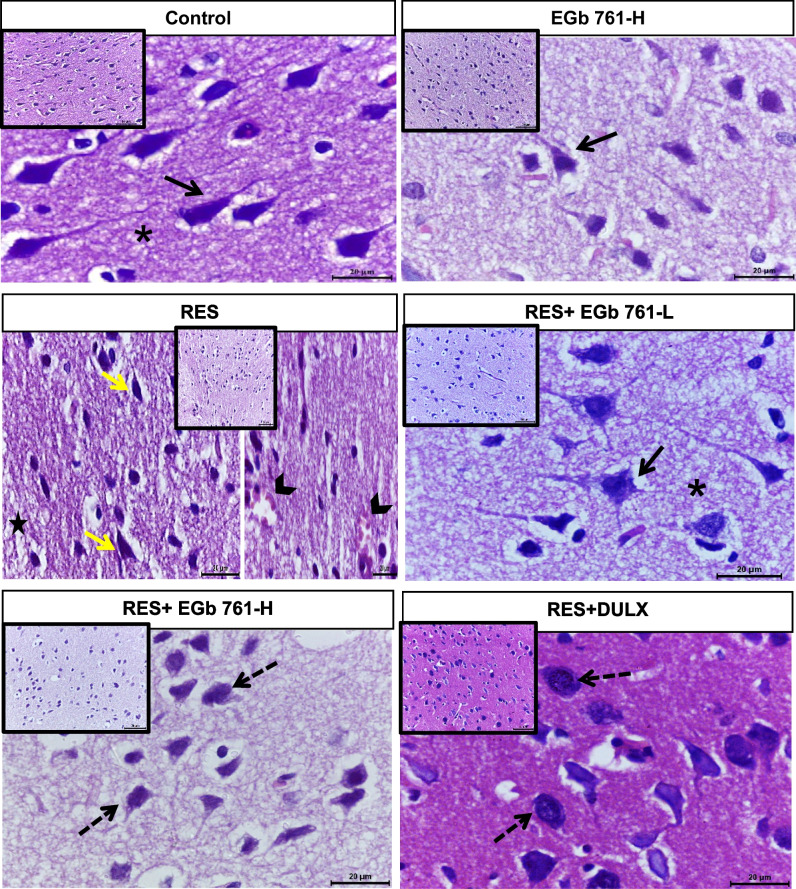
The ameliorative effect of EGb 761 on histopathological alterations in cerebral cortex of RES-injected rats. Photomicrographs showing H&E staining cortical tissue of all experimental groups (*n* = 5). Scale bar equals 20 µm for high magnification and equals 50 µm for low magnification which inserted at upper corner of images. Black arrows and asterisk refer to normal neuronal cells and intact homogenous neuropil, respectively. Yellow arrows, arrow heads, and star show necrotic pyramidal cells, infiltration of inflammatory cells, and vacuolated neuropil, respectively. Black dashed arrows indicate nerve cells with acidophilic cytoplasm

As illustrated in Fig. 7, the control group showed normal architecture of cerebral cortex with densely stained pyramidal cells with basophilic cytoplasm, normal glial, and granular cells in intact homogenous neuropil. Moreover, EGb 761-H group revealed normal neurons with more or less normal neuropil. Contrarily, RES injection provoked neuronal degeneration which was manifested by the presence of necrotic pyramidal cells having pyknotic nuclei, shrunken granular cells, glial clustering, inflammatory cell infiltration, and highly degenerative neuropil. Furthermore, both EGb 761-H and DULX administered to depressed rats partially restored morphology of pyramidal cells but with clear cytoplasmic eosinophilic alterations in slight degenerative neuropil. While EGb 761-L treatment illustrated the best ameliorative effect evidenced by non-vacuolated neuropil with normal pyramidal architecture of neuron with vesicular hyperchromatic nucleus and long axon hillock.

### EGb 761 overcame RES-related cortical synaptic loss in aged rats by increasing the immune expression of synaptophysin (synaptic protein)

**Fig. 8:**
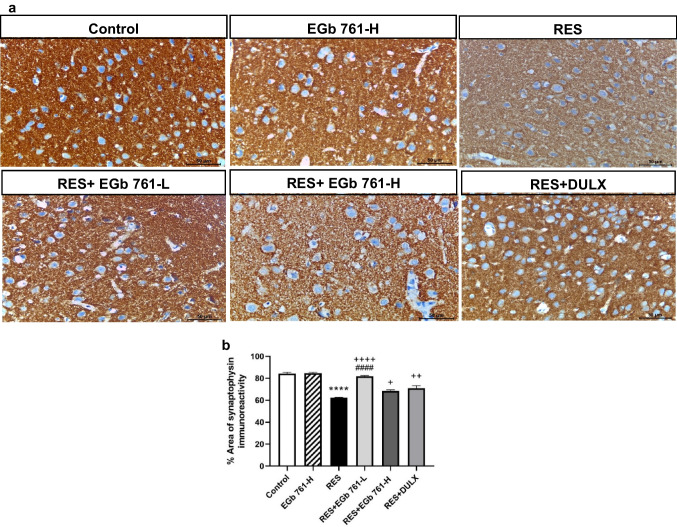
EGb 761 overcame the loss of cortical synapses elicited by RES. **a** Representative photomicrographs of synaptophysin immunohistochemistry at scale bar equals 50 µm. **b** The area percentage of synaptophysin immune reaction (*n* = 5 per group). Values in bar chart represented as the mean ± SEM. ****p < 0.0001 compared with the control group; ^**+**^*p* < 0.05, ^**++**^*p* < 0.01, ^**++++**^*p* < 0.0001 compared with the RES group; ^**####**^*p* < 0.0001 compared with the DULX group. Statistical analysis was performed by one-way ANOVA followed by Tukey’s multiple comparisons test

The control group and EGb 761-H group exhibited strong positive synaptophysin immune reaction (Fig. 8). Differently, RES injection enhanced a markedly lower percentage area of synaptophysin immunoreactivity (− 26.09%) relative to the control group. The treatment of depressed animals with EGb 761-L, EGb 761-H, or DULX showed apparently higher percentage area covered by synaptophysin immune reaction (31.31%, 9.97%, and 13.89%, respectively) in comparison with the RES group suggesting that EGb 761 especially EGb 761-L had the greatest potential to ameliorate RES-induced synaptic loss.

## Discussion

Depression in late life is common and its prevalence is elevating with population aging (Zhao et al. 2023). Although the antidepressant drugs are first option for treating LLD, their poor responsiveness and their several drawbacks limit their use among the older adults (Hsu et al. 2022; Srifuengfung et al. 2023). Thus, the amelioration and enhancement of LLD with safe and effective therapeutic agents from natural products are urgently needed. Hence, we established a depression model induced by RES injection in aged female rats to evaluate the alleviation and antidepressant effects of EGb 761 at two different doses compared with DULX as standard antidepressant drug.

RES is a valuable tool to induce depression-like phenotypes in rodents and is validated as a correlate of studying depression in humans (Zong et al. 2019; Qian et al. 2023b). Indeed, the rats injected with RES illustrated behavioral instabilities, neurobiological alterations, comparable to the symptoms which appear on patients with depression (Antkiewicz-Michaluk et al. 2014; Samad et al. 2021). In the present study, depressive-like patterns including locomotor and exploratory activities as well as hopelessness were effectively verified using OFT and FST, respectively. In the OFT, rats treated with RES revealed the reduction in the total traveled distance and elevation in the time spent in the central area. Although the increased duration spent in the central zone during the OFT has been interpreted as an anxiolytic effect in other studies (Li et al. 2017; Cai et al. 2020), in our study, this finding may be attributed to a decrease in overall activity and motivation rather than a reduction in anxiety. This could be due to the sedative effects of RES, which may cause lethargy and decreased exploratory behavior, as observed in prior studies (Khadrawy et al. 2017; Abdel-Rasoul et al. 2023). However, contradictory outcomes—a reduction in the duration of central area exploration in RES-injected rats—have also been reported. This discrepancy could be explained by differences in doses, treatment schedules, animal strains, and other experimental factors (Qian et al. 2023b, 2024).

Besides, the RES group in the current study exhibited a prolonged immobility duration in FST. The findings of OFT and FST reflected psychomotor retardation and behavioral despair in human depression, respectively, indicated that RES administration could induce depression state. These observations were supported by Zhao et al. (2022) and Park et al. (2018) who observed various depression-like behaviors following RES injection during OFT and FST. Interestingly, EGb 761 treatment markedly mitigated the depressive-like phenotypes in RES-administered rats compared to the RES group. Our results suggested that EGb 761 had prominent antidepressant efficacy. Consistent with our results, a recent study of Wang et al. (2020) illustrated that the administration of diterpene ginkgolides (main components of *Ginkgo biloba* L. leaf extract) ameliorated anxiety and depressive-like behavioral phenotype in rats’ models.

According to the monoamine theory, it is evident that the deficiency of monoamines essentially 5-HT and NE in the brain are responsible for the development of depressive-like disorders (Haase and Brown 2015; Ferrari and Villa 2017). Several studies indicated that the disruption of monoaminergic activities in cortical regions, implicated in mood regulation, represents the main mechanism triggering the pathogenesis of depression (Wei et al. 2018; Arisha 2022). Consistent with them, the chronic RES injection in our study resulted in a decrease in levels of monoamine including 5-HT and NE in cortical region. RES is a strong and irreversible blocker of the vesicular monoamine transporter-2 which suppresses monoamines reuptake and storage into the synaptic vesicles (Schossler Garcia et al. 2021). Thus, it enhances monoamines oxidative catabolism by monoamine oxidase leading to their depletion and formation hydrogen peroxide resulting in induction depression-like symptoms in experimental animals (Antkiewicz-Michaluk et al. 2015).

In contrast, EGb 761 supplementation in depressed rats significantly augmented both 5-HT and NE cortical levels which correlated with the improvement in behavioral parameters in OFT and FST compared to those in the RES group. These findings suggested that EGb 761 exerted antidepressant-like effects by activating the monoaminergic system which is similar to the action of antidepressants. Our results are in harmony with the study of Verma et al. (2020) who reported that EGb 761 elevated cortical and hippocampal 5-HT and NE in aluminum induced Alzheimer’s disease-based model.

Besides the monoamine imbalance hypothesis, it is well-known that the oxidative/nitrosative stress (ROS/RNS) is the critical pathological process in depression (Moylan et al. 2014). Evidence of a pro-oxidative condition has been confirmed in patients suffering from depression (Hassamal 2023). Indeed, brain tissues are supremely vulnerable to oxidative damage because of their high oxygen consumption, weak antioxidant capacity and high peroxidizable fatty acids content (Cenini et al. 2019). These polyunsaturated fatty acids are very susceptible to attack by free radicals. Subsequently, they activate lipid peroxidation and generate MDA as an end-product (Vaváková et al. 2015). Moreover, NO has essential roles in intracellular signaling in neurons. However, excess NO can be neurotoxic resulting in neuronal tissue damage (Nkpaa et al. 2021; Tewari et al. 2021). Consequent to increased ROS and RNS levels, depression is also associated with the reduction in antioxidants levels including GSH (Somani et al. 2022). GSH is the primary antioxidant in the brain which scavenges the free radicals, oxidizes into GSSG form and protects neurons against oxidative injury (Fisher et al. 2020).

In the present investigation, RES-injected rats exhibited marked signs of oxidative and nitrosative burden accompanied by disruption in antioxidant defense systems in cortical tissues. These are evidenced as a marked augmentation of MDA, NO, and GSSG levels with a severe depletion of GSH level. Our findings are consistent with the results from prior studies (Fahmy et al. 2022; Kuzay et al. 2022). Furthermore, it was reported that the oxido-nitrosative stress exacerbated after RES administration could be attributed to excessive production of free radicals resulting from oxidative catabolism of monoamines due to its inhibiting effect on reuptake and storage of monoamines (Khurana and Bansal 2019).

Notably, EGb 761 treatment effectively attenuated RES-stimulated cerebral oxido-nitrosative damage which inferred by the remarkable reduction of MDA, NO, GSSG levels, and elevation in cerebral antioxidant defense GSH level. Our findings confirmed the antioxidant and anti-lipoperoxidative properties of EGb 761 which came in accordance with the other research (Essawy et al. 2022). This action is linked to the chemical structure of the flavonoids in EGb 761 which have regulatory activities on ɤ-glutamyl cysteine synthetase enzyme involved in GSH synthesis. In addition, their direct effects to scavenge free radical species-triggered peroxidation and remove pro-oxidant transitional metal ions (Sadowska-Krępa et al. 2017; Di Meo et al. 2020). Thus, the antioxidant abilities of EGb 761 may participate in its antidepressant-like effects.

Undoubtedly, brain function is vastly dependent on the primary role of mitochondria in energy metabolism. Due to the high energy consumption of brain tissue, it is more vulnerable to the detrimental effects of reduced energy production (Bansal and Kuhad 2016). According to numerous studies, disturbance in brain energy metabolism plays an important role in depression propensity. In addition, cumulative research illustrated that depression is linked to mitochondrial dysfunction in different brain regions because of the potential role of mitochondria in ATP synthesis, reactive neurotransmission and plasticity (Shen et al. 2020; Jiang et al. 2024). The current findings revealed that RES injection disturbed the energy metabolism in term of depletion ATP level. Contrarily, the administration of EGb 761 particularly EGb 761-L in RES group ameliorated the adverse effect of RES on ATP production indicating that EGb 761 has mitochondria-protecting properties. Dai et al. (2014) reported that ATP synthesis declined following H2O2-induced oxidative injury in HT22 hippocampal cells. Therefore, in our study, the negative effect of RES on ATP production is likely resulting from the high free radicals’ levels created after RES injection.

One of the most significant outcomes of ROS/RNS overproduction and a reduction in ATP levels is DNA damage (dos Santos et al. 2019). Patients with depression exhibited a higher level of DNA damage (Czarny et al. 2017; Ahmadimanesh et al. 2019). Our findings illustrated a significant elevation in DNA fragmentation percentage in RES group. Interestingly, the treatment of RES group with EGb 761 obviously protected cortical tissue from genotoxicity as indicated by a reduction in percentage of DNA fragmentation. Gomaa et al. (2020) reported that EGb 761 ameliorated the increased in DNA fragmentation percentage in cisplatin-induced neurotoxicity in rats. The protective effects of EGb 761 against DNA fragmentation may be credited to its vigorous antioxidant capacity.

DNA fragmentation is an essential hallmark of cell apoptosis. Neuronal apoptosis has been well-known as a crucial mediator in the etiology of depressive disorders (Shahzad et al. 2022; Xu et al. 2023). DNA damage activates p53 expression that triggers intrinsic pathway of apoptosis which ends with the activation of caspase-3 that is executioner pro-apoptotic protein. In addition, caspase-3 is regarded as a key mediator of apoptotic processes in neuronal cells (Nkpaa et al. 2019; Bliźniewska-Kowalska et al. 2023). In the present study, the marked elevation in the cortical caspase-3 immunoreactivity was observed in RES-injected rats and was notably abated after EGb 761 treatment indicating a potential prospective role of EGb 761 to reduce neuronal apoptosis. Consistently, Adebayo et al. (2022) reported that EGb 761 supplementation suppressed executioner caspase-3 in cortico-cerebellar neurons in rotenone-induced mice model of Parkinson’s disease.

Regarding the histopathological examination, RES-injected rats in the present study revealed neuronal malformation, pyknosis, and neuropil vacuolation in the region of cerebral cortex. These findings are related to elevated oxidative stress and apoptosis in this brain region after RES injection. In contrary, concomitant administration of EGb 761 particularly EGb 761-L improved the histopathological changes in the cortical region along with modulating redox homeostasis and protecting from apoptosis. Our observations go in line with those of Zeng et al. (2018) who found that supplementation of EGb-761 significantly ameliorated degenerative changes and the shrunken cells related to permanent middle cerebral artery occlusion in cortical and hippocampal regions in rats.

Inevitably, brain morphological and cellular alterations in specific brain regions implicate synaptic dysfunction in the pathophysiology of this disorder (Leung et al. 2022). Synaptic loss and deficits in functional connectivity are supposed to trigger symptoms associated with depression. One of the key markers for synaptic density is synaptophysin (Holmes et al. 2019). Synaptophysin is a plentiful integral membrane glycoprotein of presynaptic vesicles which regulates synapse formation and is involved in neurotransmitters release (Liu et al. 2017). Depression animal model revealed the reduction in expression levels of synaptophysin in prefrontal cortex and hippocampus (Li et al. 2022; Qian et al. 2024). Similarly to these outcomes, the present study illustrated a striking reduction in the cortical synaptophysin immunoreactivity in RES group suggested a decline in synaptic density. In contrast, the treatment with EGb 761 rescued synaptic density in the cortical region following RES injection by increasing the synaptophysin immunoreactivity. The alleviating effect of EGb 761 on synaptophysin immune expression suggested its antidepressant effect which aligns with the prior studies which reported that antidepressants contributed to upregulation of synaptophysin expression (Seo et al. 2014; Qian et al. 2023a).

Overall, EGb 761-H supplementation demonstrated effects similar to those of DULX, a widely used antidepressant for the elderly, in attenuating depression-like symptoms in the RES-induced depression model in aged rats. However, EGb 761-L supplementation showed superior effects compared to both EGb 761-H and DULX, highlighting the importance of dose dependency in the therapeutic outcomes. These findings suggest that lower doses of EGb 761 may have more prominent antidepressant-like effects in this model, which may be linked to specific interactions with neurotransmitter systems or other molecular mechanisms that require further exploration.

## Conclusion

In conclusion, while the therapeutic effects of *Ginkgo biloba* L. and its potential in depression have been explored in previous studies, our study provides novel insights by specifically investigating the antidepressant effects of EGb 761 in an aged rat model of depression. Additionally, our study is unique in its focus on the role of both serotonergic and noradrenergic systems in mediating these effects, alongside the impact on oxidative/nitrosative stress. Furthermore, we addressed the gap in the literature regarding the use of EGb 761 in older subjects, who are often underrepresented in such studies. Our findings provide new evidence supporting EGb 761 as a potential alternative therapeutic option for depression in aging populations, contributing to the development of more targeted and effective treatments.

## Data Availability

All source data for this work (or generated in this study) are available upon reasonable request.

## Abbreviations

LLD: Late-life depression
5-HT: Serotonin
NE: Norepinephrine
RES: Reserpine
DULX: Duloxetine
DMSO: Dimethyl sulfoxide
EGb 761: The standardized extract of *Gingko biloba* L. leaves
i.p: Intraperitoneal
OFT: Open field test
FST: Forced swimming test
GSH: Reduced glutathione
GSSG: Oxidized glutathione
DTNB: 5,5′ Dithiobis-(2-nitrobenzoic acid)
MDA: Malondialdehyde
TBA: Thiobarbituric acid
NO: Nitric oxide
H&E: Hematoxylin and eosin
